# Towards Higher Oil Yield and Quality of Essential Oil Extracted from *Aquilaria malaccensis* Wood via the Subcritical Technique

**DOI:** 10.3390/molecules25173872

**Published:** 2020-08-26

**Authors:** M. Samadi, Z. Zainal Abidin, H. Yoshida, R. Yunus, D. R. Awang Biak

**Affiliations:** Department of Chemical and Environmental Engineering, Faculty of Engineering, University Putra Malaysia, UPM, Serdang 43400, Selangor, Malaysia; moonlight.smd@gmail.com (M.S.); yoshida.asui@gmail.com (H.Y.); robiah@upm.edu.my (R.Y.); dradiah@upm.edu.my (D.R.A.B.)

**Keywords:** *Aquilaria malaccensis*, subcritical water extraction, wood, gaharu, essential oil

## Abstract

A method that delivers a high yield and excellent quality of essential oil, which retains most of its value-added compounds, and undergoes least change after the extraction process, is greatly sought after. Although chemical free methods are acceptable, they call for an extensive processing time, while the yield and quality from these methods are often disappointing. This work utilizes subcritical water technology to address these issues. In this undertaking, essential oil was extracted from *Aquilaria malaccensis* wood by way of subcritical conditions, and characterized through gas chromatography/mass spectroscopy (GC/MS). Optimization through response surface methodology revealed temperature to be the most critical factor for the extraction process, while the optimum conditions for temperature, sample-to-solvent ratio, and time for subcritical water extraction was revealed as 225 °C, 0.2 gr/mL, and 17 min, respectively. The subcritical water extraction technique involves two simultaneous processes, which are based on good fitting to the two-site kinetic and second order model. In comparison to the hydrodistillation method, GC/MS results indicated that the quality of *A. malaccensis*’ wood oils, derived through the subcritical water technique, are of significantly better quality, while containing many constructive value-added compounds, such as furfural and guaiacol, which are useful for the production of pesticides and medicines. Pore size, functional groups, and morphology analysis revealed the occurrence of substantial damage to the samples, which facilitated an improved extraction of bio-products. In comparison to conventional methods, the use of the subcritical method not only involves a shorter processing time, but also delivers a higher oil yield and quality.

## 1. Introduction

Of late, the anti-parasitical, bactericidal, fungicidal, cosmetic, pesticide, food additive, perfumery, and medicinal possibilities of essential oils, has gained much attention globally [1]. Generally, essential oils derive from secondary metabolites with complex compositions, and the number of components may range from a dozen to several hundred [2,3]. They compose of complex mixtures of fairly hydrophobic volatile (around 100 u) to semi-volatile compounds, usually with a strong odour, rarely coloured, soluble in organic solvents, and insoluble in water [4]. Terpenoids (mono- and sesquiterpene hydrocarbons) and their oxygenated derivatives (hydroxyl and carbonyl), along with aliphatic aldehydes, alcohols, phenols, ethers, aldehydes, ketones, and esters are considered the main components of essential oils [1].

*Aquilaria malaccensis*, also known as agarwood or gaharu, is among the valuable *Aquilaria* species. *Aquilaria malaccensis* wood has many useful medicinal applications, and is used in traditional medicine to treat pain, fever, rheumatism, and asthma. Additionally, researches on non-traditional medicine have revealed that the wood of *A. malaccensis* has a remarkable anticancer activity [5]. Over recent years, the increased use of a*quilaria malaccensis* has led to over-harvesting, and its inclusion in the list of threatened trees [6]. 

The factors determining the quality of essential oils include fragrance strength (composition) and longevity, resin content, geographical origin, and oil purity. There are several ways in which the quality of essential oil can be compromised. This includes the condition of the growing plants prior to their harvesting, processing, and storage. Agarwood oil is traded worldwide, especially in the United Arab Emirates, Saudi Arabia, China, and Japan. Currently the price of high-quality agarwood oil ranges from RM200 to RM2000 per tola (12 mL) [7]. The wholesale price of high-quality agarwood oils varies from 30,000 to 50,000 USD per litre [8]. To date, no standards have been set for the quality control of essential oil. For the most part, the FDA considers essential oils either a cosmetic or a drug, depending on their intended use. The FDA makes decisions concerning the regulation of essential oils on a case-by-case basis. 

Hydro-distillation (HD) and steam distillation are the two common methods employed by industries, for the extraction of essential oil from the wood of *A. malaccensis*. However, these conventional methods have been observed to be exceedingly time-consuming [9,10]. The essential oil extraction process can extend to as long as 16 h. Other drawbacks associated to these conventional methods include the loss of volatile compounds, low extraction efficiency, degradation of unsaturated compounds, and high energy consumption [11]. While other extraction methods have been engaged to address these drawbacks, they were also found to be problematic or inefficient. For instance, the supercritical fluids extraction (SFE) method was developed, to reduce the time consumption, and also to improve the extraction efficiency (i.e., higher yield) [12]. However, this extraction method involves the emission of carbon dioxide, which is detrimental to the environment in terms of the greenhouse effect [13]. The soxhlet and accelerated solvent extraction (ASE) methods were used to improve the essential oil extraction from *A. Malaccensis* [10]. However, although both these methods appeared to improve the yield of essential oil, they are deemed undesirable due to their use of organic solvents (such as n-hexane). Organic solvents are not only toxic and hazardous, but also economically unappealing when used for the extraction of essential oil. Other methods, such as ultrasound assisted, ohmic heating, and microwave assisted extraction [14], have yet to deliver a satisfactory yield. Thus, an innovative extraction method, for addressing the problems mentioned above, remains essential.

Subcritical water extraction (SCWE), a new and promising extraction method, is safe, fast, economical, and environmentally friendly. This method entails the use of water subjected to high pressure, which raises its temperature to above its normal boiling point. The use of water, as the solvent for the extraction of essential oil, is both cost-effective and environmentally friendly [15]. Furthermore, SCWE has been observed to require a significantly shorter extraction time (around 2–3 times), and the consumption of a lower amount of raw material, to produce a higher quality and quantity of essential oil [16]. Previously, the utilization of the SCWE method, for the extraction of essential oil from *Aquilaria crassna*, under subcritical conditions (100, 125, and 150 °C), resulted in a higher yield of *A. crassna* oils in a shorter time period, in comparison to the utilization of hydrodistillation [16]. These findings suggest that in terms of time, yield, and quality, the SCWE method could represent a better alternative to the currently available conventional methods, for the extraction of *A. malaccensis*’ oil. With this in mind, this undertaking delves into the potential of employing SCWE for the extraction of *A. malaccensis* essential oil. To date, no studies have been conducted on the optimization of subcritical water extraction (SCWE) for the extraction of *A. malaccensis* oil from wood. This investigation is aimed at bridging this gap.

## 2. Materials and Methodology

### 2.1. Sample Preparation

Wood of *Aquilaria malaccensis* was collected from the Forest Research Institute Malaysia (FRIM) garden. The wood was air-dried in a dark room away from sunlight (to avoid the loss of volatiles due to light and temperature), and stored in a bag at a cool room temperature (4 °C), for the experiments to follow. All botanical materials require size-reduction before the extraction process, to improve sample distribution, heat transfer, and access to the cells’ content. This will serve to quicken the release of essential oil. A saw was used to cut the wood into smaller pieces. Then, each piece of wood was shredded with a small knife. The final step in the extraction preparation process involves the use of a grinder (Panasonic miller) to crush the shredded wood. This is to prevent the loss of volatiles. 

### 2.2. Extraction of Essential Oil

#### 2.2.1. Hydrodistillation

A Clevenger-type apparatus, described by European pharmacopeia, was used to extract essential oil from *A. malaccensis* wood. Prior to the extraction process, the wood was soaked in water for seven days, in order to speed up the release of essential oil. Then, 100 g of air-dried and size-reduced wood of *A. Malaccensis*, and an appropriate amount of distilled water, was placed in a bowl-shaped heating mantle, which was attached to the Clevenger. The sample mixtures were boiled at 100 °C and 1 atm to produce steam containing both water and essential oil. This product was condensed and collected in a vessel of the Clevenger. The extra condensed water was recycled to the flask during the extraction process. The extraction process ends with the removal of water, followed by the collection of floating essential oil, both through the bottom. The essential oil and wood sample, at optimized hydrodistillation conditions, were subsequently purified and analysed, for a later comparison with SCWE products.

#### 2.2.2. Subcritical Water Extraction 

**Figure 1:**
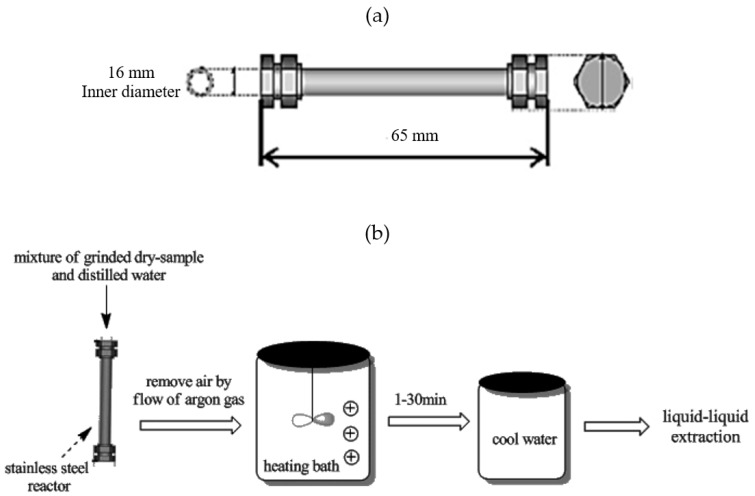
Extraction of essential oil from *Aquilaria malaccensis* by the subcritical water method: (**a**) stainless steel reactor; (**b**) subcritical water process and product recovery steps depiction.

The SCW extraction method was performed in a static-mode batch laboratory-size system (Figure 1), consisting of a 20 L heating bath (Thomas Kagaku Co., Ltd., Osaka, Japan), which employed either silicon oil (for temperatures of 100 to 170 °C) or salt of potassium nitrate/sodium nitrate (for temperatures of 180 to 271 °C) as the heating medium. 

A mixture of ground, dried raw-sample was loaded into a stainless-steel reactor (30 mL, 65 mm length, and 16 mm ID) (Swagelok, Solon, OH, USA), as shown in Figure 1. The steel reactor was then filled with water in accordance with the designated solvent-to-sample ratio. Subsequently, the reactor was surged with argon gas to remove the presence of trapped air. The cap was then secured to completely seal the reactor. Prior to the extraction, the oil bath was preheated to the desired temperature (100–271 °C). As soon as the heating bath (oil or salt bath) reached the set temperature, the reactor (filled with sample and water 0.08–0.22 gr/mL) was immersed in the bath. The bath is equipped with an agitator, which serves to distribute the heat evenly during the extraction process (Figure 1b). On completion of the extraction process (within 1–34 min), the reactor was pulled out and immediately dipped into cool water. Once the reactor was cooled down, the mixture, comprising sample and solvent, was transferred to a test tube for further purification and separation of essential oil, through the liquid-liquid extraction method, which is described in the following section.

### 2.3. Essential Oil Recovery and Yield Calculation

The liquid part of the mixture, containing water and essential oil (aqueous mixture), was poured into a separation funnel, and the liquid-liquid extraction process proceeded with the addition of 5 mL of n-hexane, for the removal of essential oil from the mixture. The liquid-liquid extraction process was replicated twice to ensure that no essential oil remained in the water. The hexane was removed with the use of a rotary evaporator. In the final step, sodium sulphate anhydrous was applied to the essential oil to remove any moisture or water. The extracted essential oils were stored in a dark-sealed-vial at 4 °C for further analysis. The yield of each essential oil was calculated with the following equation:

(1) $$y\;=\;\frac{V\times 100}{W}$$

where y is the yield of essential oil (% *v/w*), V is the volume of collected essential oil (mL), and W is the weight of the plant material (g) [5]. All experiments were carried out in triplicate, and the relative standard deviations, between replicate samples within the experimental range, were less than 5%.

### 2.4. Subcritical Water Extraction Design of Experiments and Statistical Analysis

Through screening experiments, the practical range of influential parameters (temperature, time, and solid to liquid ratio) was determined as follows: 100–271 °C for temperature, 0.08–0.2 gr/mL for sample-to-solvent ratio, and 1 to 30 min for extraction time. In this work, RSM incorporated with central composite design (CCD) at two levels was applied to ascertain the optimum value of the oil yield. Twenty-eight experiments (Supplementary Materials Table S1) were performed and analysed using ANOVA. Linear, 2FI (two factorial), quadratic, and cubic models were fitted to the experimental data to acquire the regression equation. Then, analysis of variance (ANOVA) was employed to provide the statistical analysis details (lack of fits, PRESS, and others). This is to ensure the adequacy of each model [17]. In the next step, the effect of influential factors (temperature, sample-to-solvent ratio, and time) on the yield of essential oil was analysed. The process concludes with the validation of the model. 

### 2.5. Qualitative Assessment of Bio-Products (Essential Oil and Wood Sample)

#### 2.5.1. Gas Chromatography Mass Spectroscopy (GC/MS) of Essential Oil

The extracted essential oils, from HD and SCWE, were analysed using the Shimadzu Auto Injector GC/MS, equipped with an FID detector and a BP5 capillary column. 20 µL of essential oil was diluted in 500 µL of n-Hexane as the solvent. In the next step, the mixture of essential oil and n-Hexane was injected with helium, which performed the role of carrier gas for identification. During the analysis, the running time was set for 90 min, and both the temperature and pressure were increased from 50 to 250 °C, and 37.1 to 100 kPa, respectively. 

Firstly, the chemical compounds were identified through their retention indices relative to n-alkanes indices on a HP-5 column. The retention indices were determined using the retention times of n-alkanes (C_5_-C_30_), which were analysed using the same instrument and under the same chromatographic conditions. NIST and WILEY libraries were used to identify the chemical compounds of the essential oils. Relative amount of the individual component was based on percentage compositions of the essential oil, calculated by way of a computerized integrator, using the total ion chromatograms. 

#### 2.5.2. Scanning Electron Microscopy Analysis

The dried samples of wood, before and after being subjected to the different extraction methods, were scanned with the use of a Hitachi (S-3400N) Tabletop Microscope system. The tested samples were fixed on adhesive tape, and sputtered with a thin layer of gold, before examination under a high vacuum condition, at an accelerating voltage of 5 kV.

#### 2.5.3. Fourier Transform-Infrared (FT-IR) Analysis

FTIR analysis was used to conduct the functional group examination. Initially, the wood samples were oven-dried overnight at 383 K. Next, an adequate amount of the dried powdered sample was finely mixed with KBr at a ratio of 1:100, and pressed into pellet form through a pressure of 100 kg/cm^2^. Infrared spectra were obtained by scanning the prepared pellets with a spectrometer (Thermo Nicolet FTIR). Dry air was continuously surged into the spectrometer to get rid of water vapour. FTIR spectra of the samples were recorded in the range of 400–4000 cm^−1^, and a resolution of x cm^−1^. Scanning by the FTIR spectrophotometer was at the rate of 200 scans per second. A plot of infrared radiation intensity, against wave number (known as the infrared spectrum), was recorded for the wood samples of *A. malaccensis*’, to qualitatively identify the surface functional groups of the samples. Subsequently, the spectrum of the untreated sample was compared to the spectra of the wood samples, after they were subjected to the HD and SCWE process. This is to analyse the value of the extraction method with regards to the breaking of chemical bonds, which consequently determines the effectiveness of the method, for the extraction of essential oils.

#### 2.5.4. Brunauer-Emmett-Teller (BET) Surface Area Analysis, and Barrett-Joyner-Halenda (BJH) Pore Size and Volume Analysis

Specific surface area and pore size distributions of *A. malaccensis*’ wood, before and after extraction by HD and SCWE, were measured with the use of a Micromeritics (USA), to assess the amount of nitrogen adsorption/desorption at 77 K. For measuring the surface area and pore size, the samples were degassed at 50 °C under vacuum, and subjected to a relative pressure of 0.99 atm. Then, the Brunauer-Emmett-Teller (BET) method was applied to measure the BET surface area and full adsorption isotherms of all the samples. 

Additionally, pore volume distribution was calculated based on the Barret, Joyner, and Halenda (BJH) method [18,19]. As for the case of nitrogen, the cross-sectional area is taken as 16.2 Å2/molecule. BET experiments are typically conducted to a relative pressure, denoted as P/P_0_, of approximately 0.3 at 77 K, where P_0_ is the saturation pressure [20]. At relative pressures above the point at which a N_2_ monolayer has formed on the solid, capillary condensation occurs within the pore structure of the material, such that the smaller pores are easily filled, and consecutively the larger pores are filled as the pressure is increased. Upon reaching the saturation point, the P/P_0_ is approximately 1.0, and the internal pore structure of the material contains condensed (liquid) nitrogen. The total pore volume can be calculated by assuming that the density of liquid nitrogen (LIN) in the pores is similar to that of bulk LIN.

## 3. Results and Discussion

### 3.1. RSM Design and Model Fitting for Optimization 

The experimental results obtained from 28 runs of Central composite design (CCD) are shown in Table 1. At low temperatures-short times of extraction, a lower yield was extracted, due to insufficient energy and time for effective breakage of cellulose and hemicelluloses. This circumstance impeded complete extraction as the structure of agarwood is both porous and fibrous. Temperature is a crucial parameter in the SCWE process, as it changes the polarity, dielectric constant, and viscosity of water. As such, temperature determines the capacity of water to perform as effectively as organic solvents [20,21,22,23]. The importance of temperature is clearly demonstrated in the results, when a rise in temperature significantly improved the yield. The high temperature and pressure of SCWE decreased the polarity, permittivity, density, surface tension, and viscosity of water to increase its diffusivity. An increase in the water’s diffusivity improves the mass transfer rate by enhancing the wetting of the matrix. This in turn facilitates a deeper penetration into the matrix particles, to boost the efficiency of the extraction process. 

Furthermore, the high temperature-pressure associated with SCWE not only decreases the water polarity, but also increases the pressure within the plant cells. This increase in pressure effectively ruptures the cell walls, pores, and oil glands of the plant cells to enhance the essential oil recovery process [24,25,26,27]. However, a prolonged extraction time under a high temperature leads to degradation, which reduces the oil yield [28,29]. Low yield at lowest and highest sample to water ratios may be explained by the extreme lack and excess of water, which can lead to charring and the hydrolytic effect, respectively. The high temperature and pressure associated with SCWE is significant as they (a) induce the effective rupturing of the cell wall, thus removing the main barrier to mass transfer, and (b) alter the properties of the water solvent to improve its solubility. 

Next, the experimental data were fitted to various regression models, including linear, 2F, quadratic, and cubic (Table 2). To ensure the adequacy of each model, the sequential F-test, lack of fit, and model summary statistics were obtained through the analysis of variance (ANOVA). As shown in Table 2, the 2, *p*-value for the quadratic model is less than 0.05, which renders it significant to this study. Hence, the quadratic model is deemed the model recommended through the ANOVA, while the cubic model is aliased. 

**Figure 2:**
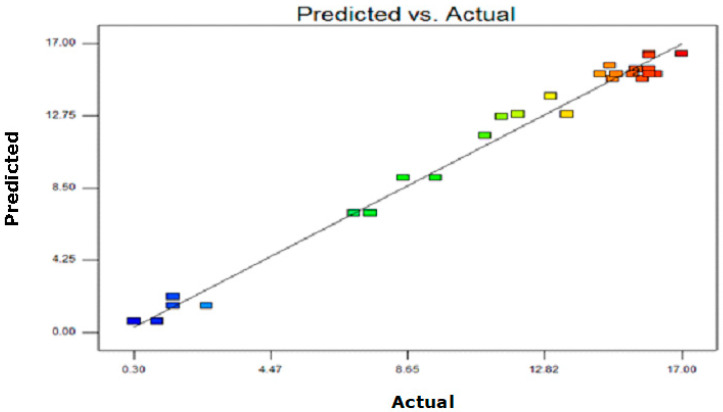
Scatter plot of predicted value versus actual value, from the central composite design for yield of *A. malaccensis* wood essential oil.

The lack-of-fit for the quadratic model (0.0778) (Supplementary Materials Table S2) was not significant, as the probability value was higher than 0.05. Standard deviation, adjusted R^2^, predicted R^2^, and prediction error sum of squares (PRESS) obtained through the ANOVA (Supplementary Materials Table S3) also supported the quadratic model. For yield of the essential oil, both the cubic and the quadratic models portrayed a low standard deviation, but the quadratic model has a lower PRESS value, as well as a high predicted, adjusted and normal R-squared. The predicted amount of *A. malaccensis* wood oil under different extraction conditions is very close to the experiment results (Figure 2). Additionally, all the points in the residual plot of experiment data (Supplementary Materials Table S4) are close to the centre line, which is an indication of satisfactory residuals. Regression analysis for fitting the quadratic model to the data of the yield of the essential oil could explain 98.6% of the yield variation (Table 3). The model’s *p*-value is less than 0.05, while the F-value is higher than the model’s mean square. This is an indication of conformation to the quadratic model [30]. Furthermore, the values of adjusted R^2^ (0.979) and precision residual sum of square (PRESS > 4) also confirmed the significance of the quadratic model.

The *p*-values were then considered to assess the significance of each variable (Table 3). The *p*-values of all variables were observed to be significant, except for the interactions between sample-to-water ratio with both temperature and time. Among the tested variables and their interactions, time-temperature interaction, time square, ratio square, and temperature square were found to have negative coefficients. The negative coefficients, which reflect the negative association between the parameters and the yield, may be explained by the possible degradation of essential oil, at very high temperatures, and prolonged extraction times. The use of the quadratic model to explain the relationship between the observed oil yield and the independent variables can be expressed as follows:

(2) $$\begin{array}{cc} Yield=15.21+ & 5.16X_{1}+0.89X_{2}+1.36X_{3}+0.14X_{1}X_{2}-2.11X_{1}X_{3}+0.29X_{2}X_{3}\\ & -3.64X_{1}^{2}-1.42X_{2}^{2}-0.41X_{3}^{2} \end{array}$$

where *X*_1_ = temperature, *X*_2_ = wood of *A. malaccensis* to water ratio, and *X*_3_ = extraction time. 

**Figure 3:**
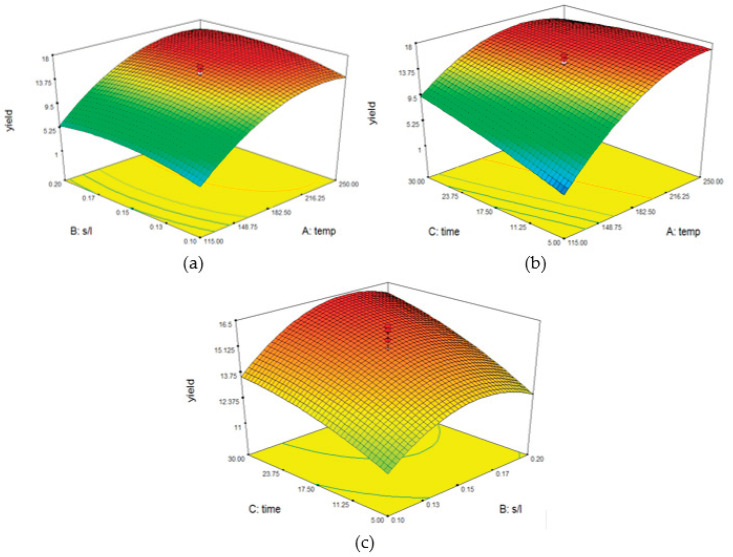
3D response surfaces for investigations on the interactive effect of variables with (**a**) denoting the temperature versus sample-to-solvent ratio, (**b**) denoting the time versus temperature ratio, and (**c**) denoting the time versus sample-to-solvent ratio, with regards to the extraction of essential oil from *A*. *malaccensis* wood.

Equation (2) was used to plot 3D response surfaces for investigations on the interactive effect of variables (Figure 3). In each of these situations, two independent variables are included, while the other remained constant. Figure 3a shows the effect of temperature and sample-to-solvent ratio on the yield of A. *malaccensis* wood oil, with the extraction time maintained at 17.5 min. Both the temperature (up to 250 °C) and sample-to-water ratio were positively associated to the yield of the essential oil. The results indicate that when the amount of sample and water is sufficient, and the temperature is raised to 250 °C, more essential oil is extracted from the wood. This is mainly due to the fact that this situation brings about a reduction in the polarity of the water. However, a further increase in temperature led to a decrease in essential oil yield, caused by adverse reactions such as hydrolysis, charring, and degradation [24,25,29]. The results also provided evidence that the sample-to-solvent ratio only has a minor effect on the yield of essential oil from wood. At ratios higher and lower than 0.12–0.2 gr/mL, the oil yield decreased due to charring or the hydrolytic effect, caused by the lack or excess of water, respectively. The optimum temperature-sample and solvent ratio was observed to be 190–250 °C and 0.13–0.2 gr/mL, respectively.

Figure 3b portrays the effect of both temperature and extraction time on the yield of A. *malaccensis* wood oil with 0.15 gr/mL of sample-to-solid ratio. The findings provide evidence that the rise in temperature brings about a higher yield of essential oil, while the extraction time has a somewhat minor effect on the yield, especially at temperatures above 200 °C. The results reveal that the parameter with the greatest impact on *A. malaccensis* wood oil yield is temperature. At higher temperatures, the mass-transfer of the solvent and plant matrix tends to increase with the decrease in the density and viscosity of water [31]. The highest yield was obtained when the temperature was around 200–260 °C, and the extraction time was equal to, or greater than 5 min. 

Figure 3c depicts the effect of both extraction time and the sample-to-solvent ratio at 182.5 °C. It was observed that an increase in both parameters led to a climb in the yield of essential oil. The greatest yield of essential oil was obtained when the sample-to-solvent ratio was in the range of 0.15–0.2 gr/mL, and the extraction time was equal and greater than 17 min. As portrayed in Figure 3a,b, low water polarity, brought about by an ideal temperature and short processing time, improves the yield of essential oil extraction. The results from response surface analysis and regression analysis verified that in terms of subcritical water extraction of *A. malaccensis* wood essential oil, temperature is the most influential factor, followed by time and sample-to-water ratio.

Model validation was performed at optimum conditions, with RSM at 225 °C for 13 min, and a wood-to-water ratio of 0.2 gr/mL, to deliver a predicted yield 16.999 µL. The extraction of essential oil from *A. malaccensis* wood, under optimum conditions, resulted in a relatively high yield of 16 µL. This validated the effectiveness of the model as the experimental and predicted values were close to similar (minor relative error oil yield = 0.699). Thus, it can be surmised that the empirical model derived from the RSM experimental design can adequately describe the relationship between the independent variables and the response of the system.

### 3.2. Qualitative Assessment of Essential Oil and Wood Sample

Both essential oil and wood samples from HD and SCW treatments were analysed to elucidate the effectiveness of different extraction methods, in terms of essential oil quality and morphological/structural changes. GC/MS was used to analyse essential oils extracted at optimum conditions for both HD (100 °C, 0.1 g/mL, 16 h) and SCWE (156 °C, 0.2 g/mL, 25 min), while raw and treated wood samples were analysed with the utilization of SEM, FTIR, and BET, to ascertain the morphology, chemical structure, surface area, and porosity of the sample. 

#### 3.2.1. Gas Chromatography/Mass Spectroscopy of Essential Oil

**Figure 4:**
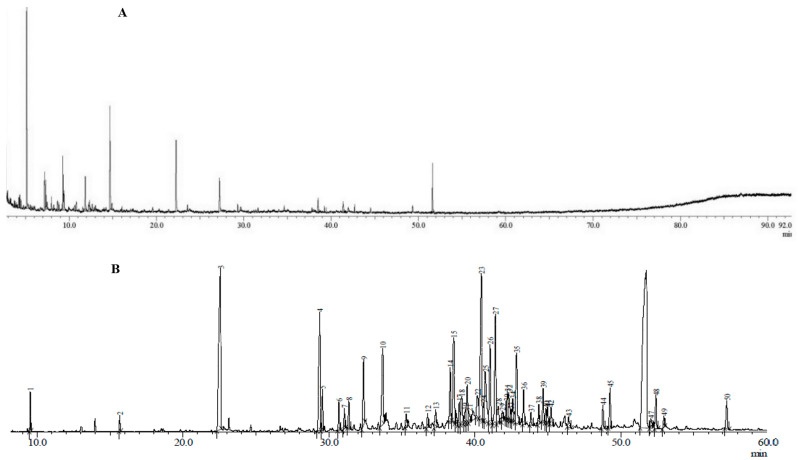
GC/MS chromatogram of *A. malaccensis* wood essential oil extracted by (**A**) SCWE and (**B**) Hydrodistillation.

Gas chromatography/mass spectroscopy (GC/MS) was used to examine the chemical compositions of *A. malaccensis* wood essential oils, extracted through HD and SCWE, at optimum conditions (Figure 4). Forty-three chemical components, constituting 92% of dark greenish *A. malaccensis* wood essential oil, extracted by way of HD were identified, while for SCWE, 50 chemical components, constituting 96% of light orange/brownish *A*. *malaccensis* essential oil were identified (Table 4). The GC/MS identified n-hexadecenoic acid (17.238%), 2-butanone, 4-phenyl (10.732%), and agarospirol (7.618%) as the major compounds of essential oil extracted by way of hydro-distillation from the wood of *A. malaccensis*, while furfural (14.36%), guaiacol (13.504%), and 2-butanone, 4-phenyl (12.042%) were identified as the major compounds of essential oil derived through SCWE.

*A. malaccensis* wood essential oil extracted through HD (1-week soaking + 16 h) and SCWE (13 min) were observed to hold common important compounds of agarwood, including hexadecenoic acid and benzylacetone. However, as depicted in Table 4, the chemical composition of *A. malaccensis* wood oil extracted by way of SCWE also contained several compounds of medicinal value (for instance guaiacol, furfural, vanillin, phenylacetaldehyde, and syringol, among others), and some low molecular weight compounds (C_5_ series, e.g., furfural and acetylfuran), which are non-existent in the oil extracted by way of HD. Moreover, as the essential oil extracted by SCWE contains a higher percentage of sesquiterpenes and oxygenated compounds, which are the quality-determinant compounds for *A. malaccensis* wood oil [7,32,33], SCWE essential oil can be considered to be of higher quality than the essential oil extracted through HD. The GC/MS results are in agreement with the results from previous studies [15,34,35,36], which reported that *A. malaccensis* wood essential oil contains important signature compounds, such as agarospirol, hexadecenoic acid, and benzylacteone, among others.

**Figure 5:**
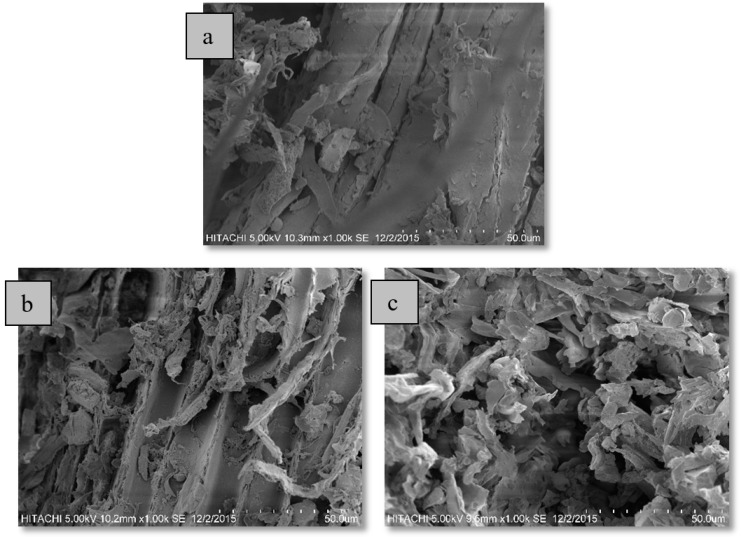
Micrographs of *A. malaccensis* wood (**a**) before, (**b**) after HD, and (**c**) after SCWE.

The increase in the number of compounds in the oil extracted through SCWE, during a significantly short time, can be explained by the low polarity, permittivity, viscosity, and surface tension of water, which resulted in the enhancement of water efficiency, with regards to the extraction of different classes of compounds [15]. The high pressure-temperature condition of SCWE enables the water to emulate the performance of organic solvents, and improve the solubility of compounds [25,27,33]. For instance, compounds such as furfural and guaiacol, which are slightly soluble in water at ambient temperature [37], tend to increase in solubility at an elevated temperature and pressure [23,27]. As shown in Figure 5, high temperature-pressure not only facilitates the effective breakage of a plant’s cell-wall [38,39], but also facilitates the degradation of lignin, xylose, cellulose, and hemicelluloses during the subcritical process. This is made evident through the appearance of small compounds such as furfural, syringol, vanillin, guaiacol, and its derivatives [40,41]. Due to the fact that the performance of SCWE occurs at high temperatures and pressures in the presence of water, unwanted reactions such as hydrolysis, degradation, charring, and others are inevitable. 

Differences between the results of this study and other findings, in terms of identified chemical compounds, may be attributed to the different extraction systems (HD and SCWE), natural sample variations due to their different regions of origin, and different inoculation methods for the production of resinous wood [34,42]. It has been established that the amount of essential oil, as well as the chemical composition of essential oil, varies according to the extraction method employed [43]. The difference in percentage of chemical compounds in the essential oils extracted by HD and SCWE during this study is to be expected, considering the nature of their extraction conditions. Given that the total number of identified compounds increased during SCWE, the percentage of each compound, which is calculated by considering the total compounds, is anticipated to decrease. 

As mentioned previously, the main compounds identified in SCWE essential oil are furfural, guaiacol, and 2-butanone, 4-phenyl, which have many applications. Furfural (C5H4O2) is a heterocyclic aldehyde and a natural main product of xylose or lignocellulose decomposition [44,45]. Furfural is known for its biological activities, toxicity, and antibacterial activity [46]. Guaiacol (C_7_H_8_O_2_), meanwhile, is an important phenolic compound, with a strong antioxidant activity [47,48]. Guaiacol is also known to have potent anti-inflammatory and anti-cancer activities [47]. 2-butanone, 4-phenyl (also known as benzylacetone) is a ketone that is used as additives in the production of cosmetics, soap, perfume, and food [49]. It is also an important pharmacologically bioactive compound, which explains the use of *A. malaccensis* wood in traditional medicine [50]. 

#### 3.2.2. Scanning Electron Microscope (SEM) Analysis of Wood Sample

The micrographs of untreated *A*. *malaccensis* wood (i.e., the control sample) and *A*. *malaccensis* wood after HD and SCWE extraction is exhibited in Figure 5a–c, respectively. The micrographs of *A*. *malaccensis*’ wood clearly show significant changes in the structure of the sample, after HD and SCWE extraction. However, with subcritical conditions, the sample’s cell walls were severely ruptured and collapsed, due to the elevated temperature and pressure. The disruption of the cell walls promotes the efficiency of the water, by removing the barrier in its way. In addition, the increase in pressure within the plant glands, serves to improve the essential oil extraction process [26,27,43,51]. Previous investigations also reported severe rupturing of the sample‘s cell walls, following SCWE at different temperatures [39,52]. The SEM analysis also provided evidence that despite the long duration of the HD process (one-week soaking followed by 16 h of extraction), the hydro-distillation extraction process failed to completely extract essential oil from the plant cells. This can be attributed to the fact that the *A. malaccensis*’ wood glands were only partially ruptured (Figure 5b). The inability of HD, in comparison to other new extraction methods, to efficiently extract essential oil, has been reported by other researchers [43,51,52]. 

The SEM results verified our hypothesis that SCWE improves oil extraction through (i) the rupturing of cell walls and (ii) the modification of the properties of water as the solvent. *A. malaccensis* wood is made up of a structure comprising lignin, cellulose, and hemicellulose. Cellulose is a long chain of glucose units connected by β(1, 4) glycosidic linkages. The mechanical strength and chemical stability of these long chains stem from the existence of intra- and inter-molecular hydrogen bonds between them. However, with SCWE, the high temperature and pressure reduces the polarity of water to render its behaviour similar to an organic solvent. The weakening of water polarity also disturbs the intra- and inter-molecular hydrogen bonds between the chains, making them fragile and considerably susceptible to cell wall rupture. This facilitates the deeper penetration of solvents into the plant matrix during the extraction process. Simultaneously, the density and viscosity of water is reduced to promote diffusivity, and boost the mass transfer of essential oil into the solvents for better extraction efficiency [26,31]. The SEM images also suggest more losses of structural water in the wood due to evaporation during hydrolysis process at SCWE conditions compared to HD. 

#### 3.2.3. Fourier Transform Infrared Spectroscopy Analysis of Wood Sample

**Figure 6:**
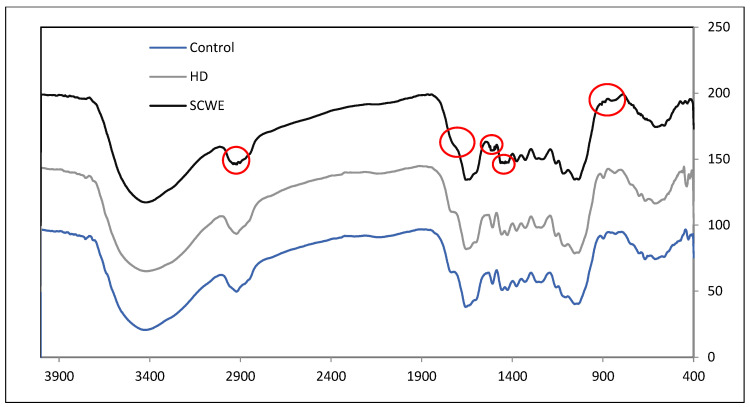
FTIR result for *A. malaccensis* wood before and after extraction by HD and SCWE.

Fourier transform infrared spectroscopy (FTIR) was used to assess the effect of SCWE on the chemical structure of *A. malaccensis* wood. The FTIR spectra of untreated *A. malaccensis* wood (the control sample), following HD and SCWE extraction, are depicted in Figure 6. The three spectra were compared within a range of 400–4000 cm^−1^. The spectrum of untreated *A. malaccensis* wood (control sample) was identical to the spectrum of the wood after HD extraction. The similarity of these spectra, considering the SEM result, indicates that hydro-distillation, despite its long duration (16 h), failed to break down the lignin, cellulose, hemicellulose, and insoluble starch to extract carbohydrate compounds. Therefore, both the FTIR and the SEM results verify the inability of HD to effectively break down the cell walls of *A. malaccensis* wood and extract its essential oil. This result explains the lower yield of essential oil extracted through HD, in comparison to SCWE. 

FTIR spectrum of *A. malaccensis* wood after SCWE was observed to be slightly different, from the control and HD spectrum. The intensity of the absorbance bands for cellulose and hemicellulose [43,50] either decreased a little, or vanished entirely in the SCWE spectrum’s points of 885 cm^−1^ (C-H bend aromatic/deformation vibration), 897 cm^−1^ (C-O-C stretching), 1030 cm^−1^, 1170 cm^−1^ (C-O stretch vibration), 1375 cm^−1^, 1480 cm^−1^ (C-H bend), 1420 cm^−1^(CH_3_ bend), 1710 cm^−1^ (C=O stretch vibration), and 2910 cm^−1^ (C-H stretch). FTIR absorption peak of 897 cm^−1^ generally corresponds to β (1→4)-glycosidic linkages [53]. The absence of this peak (897 cm^−1^) upon treatment of SCWE (at 225 °C) is believed to be due to the breaking of β (1→4)- glycosidic bonds (cellulose to glucose) [53]. This glucose is then further decomposed into furfural as can be detected by GC/MS results presented previously. Previous workers have demonstrated the use of temperature as high as 250 °C in order to observe significant changes in FTIR of the cellulose sample [53]. 

The slight difference between the spectrums of the sample after SCWE and the control sample provides some evidence of SCWE’s ability to break down the chemical structures of cellulose and hemicellulose in the wood matrices of *A. malaccensis*. Although the changes in FTIR of the sample from SCWE in this work is not apparent, previous studies have demonstrated the effective influence of the SCWE condition on the hydrolysis of the sample’s cellulose and hemicellulose to glucose [39,53]. In short, the FTIR analysis showed some changes and partially supports other findings (yield and SEM) from this work.

#### 3.2.4. Brunauer-Emmett-Teller (BET) Surface Area Analysis and Barrett-Joyner-Halenda (BJH) Pore Size and Volume Analysis

**Figure 7:**
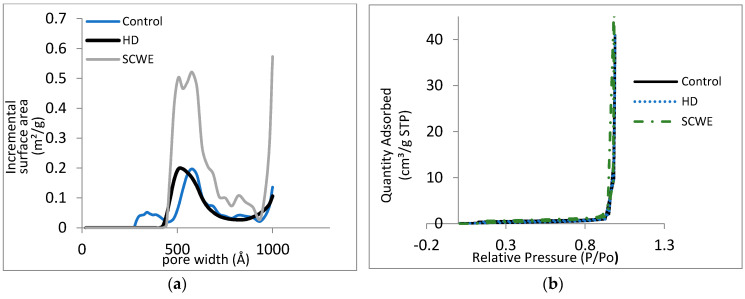
(**a**) Pore size and (**b**) isotherm linear plot of *A. malaccensis* wood before and after extraction by HD and SCWE.

The effectiveness of extraction methods on the wood of *A*. *malaccensis* was assessed through BET surface area and BJH pore size. These tests measure the surface area of the sample, as well as its pore size, by referring to the amount of the N_2_ gas they absorb. As shown in Figure 7, the pore size graph of the untreated sample (raw wood sample) is slightly different from that of the wood after extraction by HD and SCWE, in both range and distribution of pore sizes. The pore sizes in the raw wood sample in the range of 300–450 Å vanished following the HD extraction. The pore size of both HD (soaking and extraction) and SCWE changed to 400–950 Å, implying that the rupturing of small pores rendered them larger. 

The similarity of the effect of HD and SCWE on the porosity of the wood sample indicates that SCWE (similar to HD) can effectively extract essential oil, even if the extraction time is brief. However, despite the fact that the range of pore size is similar in both samples after HD and SCWE, the BET result showed that the pore volume of the wood sample significantly increased from 0.063353 cm^3^/g (i.e., before the extraction) to 0.186294 cm^3^/g after extraction by SCWE, while the increase was only 0.063172 cm^3^/g after extraction by HD. Thus, it can be surmised that even though the pores were enlarged through HD, deeper penetration into the pores was not achieved. The similarity in pore volume of the raw sample, and the sample after HD, reveals the ineffectiveness of HD compared to SCWE, when it comes to penetrating the sample for the extraction of essential oil. Consequently, based on the BET result, SCWE is not only capable of enlarging the pore size, but also effective when it comes to increasing the pore volume of the wood sample. As mentioned earlier, the changes in physical properties of water in SCWE’s high-temperature-pressure condition plays a key role in improving the extraction and mass transfer process [15,38,39]. Firstly, the high temperature-pressure of the SCWE process causes major rupturing of the cell wall, and secondly, it simultaneously decreases the viscosity and surface tension of water, which facilitates better matrix penetration, and consequently better oil liberation [15,26,27,39]. A scrutiny of the isotherm linear plots of *A. malaccensis* wood (Figure 7), before and after extraction by both HD and SCWE, revealed that they follow the same IUPAC type. The BET and BJH results confirmed that the SCWE method is more effective than the HD method, as in a short time, both pore size and pore volume of the wood sample changed significantly after SCWE. 

## 4. Conclusions

*A*. *malaccensis*’ oil was extracted from its wood through SCWE. Temperature was identified as the most significant parameter as dramatic changes in oil yield was observed during variations in temperature. The interaction between temperature and reaction time greatly influences the essential oil yield through SCWE. Based on the GC/MS results, the *A. malaccensis* wood oil extracted by way of SCWE is of significantly better quality than that extracted through hydrodistillation. Several value-added compounds with medicinal values (such as furfural and guaiacol) were observed to be present in essential oil extracted through SCWE. Further analysis through FTIR, SEM, and BET/BJH revealed that the severe damage to cell walls, cellulose, and hemicellulose of wood facilitates greater essential oil recovery during the extraction process. In short, not only is SCWE a superior method for extracting a higher yield and better quality of essential oil in a shorter time, but it also used a smaller sample volume, in comparison to conventional hydrodistillation. 

## Figures and Tables

**Table 1:** Central Composite Design (CCD) for the extraction of essential oils from *A. malaccensis* wood, by way of the Subcritical Water (SCW) method.

Run	X_1_ (°C)	X_2_ (gr/mL)	X_3_ (min)
1	115	0.1	5
2	115	0.1	5
3	250	0.1	5
4	250	0.1	5
5	115	0.2	5
6	115	0.2	5
7	250	0.2	5
8	250	0.2	5
9	115	0.1	30
10	115	0.1	30
11	250	0.1	30
12	250	0.1	30
13	115	0.2	30
14	115	0.2	30
15	250	0.2	30
16	250	0.2	30
17	93.67	0.15	17.5
18	271.33	0.15	17.5
19	182.5	0.08	17.5
20	182.5	0.22	17.5
21	182.5	0.15	1.05
22	182.5	0.15	33.95
23	182.5	0.15	17.5
24	182.5	0.15	17.5
25	182.5	0.15	17.5
26	182.5	0.15	17.5
27	182.5	0.15	17.5
28	182.5	0.15	17.5

Note: X_1_ (temperature, °C), X_2_ (sample to water ratio, gr/mL), X_3_ (time, min).

**Table 2:** Statistical parameters of the polynomial models.

Model	*F*-Value	*p*-Value
Linear	18.198	<0.0001
2-Factor interaction	2.876	0.0604
Quadratic	87.562	<0.0001
Cubic	0.407	0.8004

Note: (a) Model *F*-value is significant at “Prob > *F*” less than 0.05.

**Table 3:** ANOVA and regression coefficient for the reduced quadratic model.

Source	Sum of Squares	Degree of Freedom	Mean Square	*F* Value	*p*-Value
Model	809.679	9	89.964	142.108	<0.0001 ^a^
A—Temperature; B—Solid to Solvent Ratio; C—Time					
A ^a^	518.955	1	518.955	819.746	<0.0001
B ^b^	15.256	1	15.256	24.098	0.0001
C ^c^	36.140	1	36.140	57.087	<0.0001
AB	0.302	1	0.302	0.477	0.4982
AC	71.402	1	71.402	112.787	<0.0001
BC	1.322	1	1.322	2.089	0.1655
A^2^	97.454	1	97.454	153.93	<0.0001
B^2^	14.775	1	14.775	23.338	0.0001
C^2^	1.218	1	1.218	1.924	0.1823
Residual	11.395	18	0.633		
Lack of fit	5.681	5	1.136	2.585	0.0778 ^b^
Pure error	5.713	13	0.439		
Corrected total	821.074	27			
R^2^		0.986	Standard Deviation		0.795
Adjusted R^2^		0.979	Mean		11.414
Predicted R^2^		0.963	Coefficient of variation %		6.970
Adequate Precision		33.118	PRESS ^c^		30.185

Note: ^a^ Model *F*-value is significant at “Prob > *F*” less than 0.05, ^b^ lack of fit value is not significant relative to pure error, ^c^ PRESS is predicted residual error of sum of squares.

**Table 4:** Chemical composition of essential oil from *Aquilaria malaccensis*’ wood.

Component Name	%Presence		R.t (min)	RI
	HD	SCWE		
Butanal, 2-methyl-			2.531	643
2-Pentanone			2.733	666
2,3-Pentanedione/(Acetylpropionyl)		0.64	2.781	676
Oxiran, tetramethyl-			3.980	686
Acetylbutyryl		0.49	4.228	755
Cyclopentanone		0.402	4.330	780
Furfural		14.36	5.153	830
Acetoxyacetone		0.391	5.985	840
2-Methyl-2-cyclopentenone		0.32	7.224	880
Valerolactone<gamma->		2.041	7.333	886
2-Acetylfuran		1.516	7.499	890
2,4-Pentanedione, 3-methyl-		0.706	7.971	897
2-furylacetone		1.066	8.830	919
Furfural <5methyl->		4.011	9.312	960
Benzaldehyde	0.923	2.019	9.531	995
2-Cyclopenten-1-one, 2,3-dimethyl-		0.527	10.630	1001
Cyclotene		1.423	11.850	1006
2-Acetyl-5-methylfuran		0.569	12.244	1010
Pyrazole-4-carboxaldehyde, 1,5-dimethyl-		0.618	12.690	1047
Phenylacetaldehyde		0.552	12.926	1049
1-(5-Methyl-2-furyl)-2-propanone		0.336	13.805	1056
Acetophenone	0.852		13.976	1029
Guaiacol		13.504	14.722	1063
Benzaldehyde dimethyl acetal	0.748		15.670	1080
Mequinol		0.64	16.047	1180
Creosol		0.709	19.573	1181
Verbenone, (L)		0.4	20.586	1199
2-Butanone, 4-phenyl-	10.732	12.042	22.563	1228
4-phenyl-2-butanol	0.871		23.148	1254
Guaiacol <4-ethyl->		0.881	23.556	1245
Benzene, 1-chloro-2-dimethoxymethyl-	0.784		24.649	1260
Guaiacol <4-vinyl->		0.922	25.493	1277
Syringol		4.02	27.215	1309
4-Ethylphenyl acetate	4.713	1.11	29.361	1273
Lactic acid, 3-phenyl-, methyl ester	1.281		29.558	1421
Vanillin		1.386	29.681	1357
Guaiene Alpha	0.925	1.248	30.686	1426
gamma Elemene	0.844		31.054	1430
beta-Selinene	1.053	0.308	31.375	1454
Isoeugenol	0.204	1.158	31.694	1439
Humulene alpha	0.198	0.12	31.721	1470
5-Hydroxy-5-isopropenyl-2-methylcyclohexyl acetate	0.536		32.155	1474
beta agarofuran	2.845		32.384	1474
Anisylacetone	0.138	0.716	32.410	1462
Guaiene delta	3.355	1.427	33.689	1490
Bicyclogermacrene	0.531		33.910	1494
gamma.-Himachalene	0.288	0.122	34.966	1499
4a-Methyldecahydro-1-naphthalenyl acetate	0.612		35.297	1503
Caryophyllene oxide	0.523	0.34	36.770	1507
Spathulenol	0.844	0.72	37.328	1536
Eugenol <methoxy->	0.121	0.381	37.880	1600
Rosifoliol	2.287		38.320	1595
10-epi-gama-eudesmol	3.298	2.312	38.556	1599
gamma.-Eudesmol	1.974	0	38.743	1626
Valerianol	0.979	0.29	38.941	1633
viridiflorol	1.015	0.61	39.099	1636
beta-Eudesmol	1.594	0.421	39.486	1637
Agarospirol	7.618	3.52	40.184	1639
Postogol	1.405		40.454	1651
α-Eudesmol	1.887	0	40.723	1652
Eudesmol<dihydro->	3.067	0	41.051	1661
Bulnesol	4.882	2.103	41.410	1666
2,2,7,7-Tetramethyltricyclo [6.2.1.0(1,6)]undec-4-en-3-one	0.995		42.170	1730
Glaucyl alcohol	0.836		42.336	1732
Aristolone	0.775		42.602	1746
γ-costol	2.635	1.104	42.862	1752
Oxo-agarospirol	1.542	0.491	44.680	1822
valerenic acid	1.606	0.522	49.278	1843
Hexadecanoic acid	17.238	10.104	51.752	1935
9-Octadecenal, (Z)-	1.356	0.56	52.415	1977
Octadecanal	1.249	0.44	57.269	2000
Unidentified	7.841	3.382		
Total	92.159	96.618		

## Supplementary Materials

The following are available online: **Figure S1**. Residual plot of runs from central composite design for yield of *A. malaccensis* wood essential oil, **Table S1**. Central Composite Design (CCD) of experiments for extraction of essential oils from *A. malaccensis* wood by Subcritical water method, **Table S2**. Lack of Fit Tests, **Table S3**. Model Summary Statistics, **Table S4**. The actual and predicted values of the yield in model of extraction essential oil from wood of A. *malaccensis*.
