# Supplementary material for: Towards Higher Oil Yield and Quality of Essential Oil Extracted from Aquilaria malaccensis Wood via the Subcritical Technique
Source: Molecules. 2020 Aug 26;25(17):3872. doi: 10.3390/molecules25173872 (PMC7503260; doi:10.3390/molecules25173872)
Supplement: Supplementary file 1 [file molecules-25-03872-s001.pdf]

molecules

MDPI

Supplementary Materials

Towards Higher Oil Yield and Quality of Essential Oil Extracted from *Aquilaria malaccensis* Wood via the Subcritical Technique

M. Samadi, Z. Zainal Abidin *, H. Yoshida, R. Yunus, and D.R. Awang Biak

Department of Chemical and Environmental Engineering, Faculty of Engineering, University Putra Malaysia, 43400 UPM Serdang, Selangor, Malaysia; moonlight.smd@gmail.com (M.S.); yoshida.asui@gmail.com (H.Y.);robiah@upm.edu.my (R.Y.); dradiah@upm.edu.my (D.R.A.B.)

* Correspondence: zurina@upm.edu.my

Received: 17 July 2020; Accepted: 17 August 2020; Published: 24 August 2020

Abstract: A method that delivers a high yield and excellent quality of essential oil, which retains most of its value-added compounds, and undergoes least change after the extraction process, is greatly sought after. Although chemical free methods are acceptable, they call for an extensive processing time, while the yield and quality from these methods are often disappointing. This work utilizes subcritical water technology to address these issues. In this undertaking, essential oil was extracted from *Aquilaria malaccensis* wood by way of subcritical conditions, and characterized through gas chromatography/mass spectroscopy (GC/MS). Optimization through response surface methodology revealed temperature to be the most critical factor for the extraction process, while the optimum conditions for temperature, sample-to-solvent ratio, and time for subcritical water extraction was revealed as 225 °C, 0.2 gr/mL, and 17 min, respectively. The subcritical water extraction technique involves two simultaneous processes, which are based on good fitting to the two-site kinetic and second order model. In comparison to the hydrodistillation method, GC/MS results indicated that the quality of *A. malaccensis'* wood oils, derived through the subcritical water technique, are of significantly better quality, while containing many constructive value-added compounds, such as furfural and guaiacol, which are useful for the production of pesticides and medicines. Pore size, functional groups, and morphology analysis revealed the occurrence of substantial damage to the samples, which facilitated an improved extraction of bio-products. In comparison to conventional methods, the use of the subcritical method not only involves a shorter processing time, but also delivers a higher oil yield and quality.

Keywords: *Aquilaria malaccensis*; subcritical water extraction; wood; gaharu; essential oil

Table S1. – Central Composite Design of experiments for extraction of essential oils from *A. malaccensis* wood by SCWE method.

Run	A(°c)	B(gr/ml)	C (min)
1	115	0.1	5
2	115	0.1	5
3	250	0.1	5
4	250	0.1	5
5	115	0.2	5
6	115	0.2	5
7	250	0.2	5
8	250	0.2	5
9	115	0.1	30
10	115	0.1	30
11	250	0.1	30
12	250	0.1	30
13	115	0.2	30
14	115	0.2	30
15	250	0.2	30
16	250	0.2	30
17	93.67	0.15	17.5
18	271.33	0.15	17.5
19	182.5	0.08	17.5
20	182.5	0.22	17.5
21	182.5	0.15	1.05
22	182.5	0.15	33.95
23	182.5	0.15	17.5
24	182.5	0.15	17.5
25	182.5	0.15	17.5
26	182.5	0.15	17.5
27	182.5	0.15	17.5
28	182.5	0.15	17.5

Table S2. – Lack of fit tests.

Source	Sum of Squares	Degree of Freedom	Mean Square	*F Value*	*p-value* Prob > F	Remarks
Linear	245.008	11	22.273	50.680	< 0.0001	-
2FI	171.981	8	21.497	48.915	< 0.0001	-
**Quadratic**	**5.681**	**5**	**1.136**	**2.585**	**0.0778**	**Suggested**
Cubic	4.494	1	4.494	10.225	0.0070	Aliased
Pure Error	5.713	13	0.439	-	-	-

Table S3. Model summary statistics.

Source	Standard Deviation	R-Squared	Adjusted R-Squared	Predicted R-Squared	PRESSa
Linear	3.232	0.694	0.656	0.600	328.075
2FI	2.908	0.783	0.721	0.684	259.295
Quadratic	0.795	0.986	0.979	0.963	30.185
Cubic	0.853	0.987	0.976	0.719	230.070

a: level of fitness between the model and each point

Table S4. The actual and predicted values of the yield in model of extraction essential oil from wood of *A. malaccensis*

Run	A(°C) Temp	B(gr/ml) Solvent to Solid Ratio	C (min) Time	yield (µl)	
				Actual	Predicted
**1**	**95.0**	**0.15**	17.5	1.5	2.112
2	182.5	0.15	17.5	15.53	15.212
3	271.0	0.15	17.5	14.8	15.703
4	182.5	0.08	17.5	11	11.592
5	182.5	0.22	17.5	13	13.923
6	182.5	0.15	1.0	11.5	12.714
7	182.5	0.15	34.0	16	16.301

Note: X_1 (temperature, °c), X_2(sample to water ratio, gr/ml), X_3 (time, min)

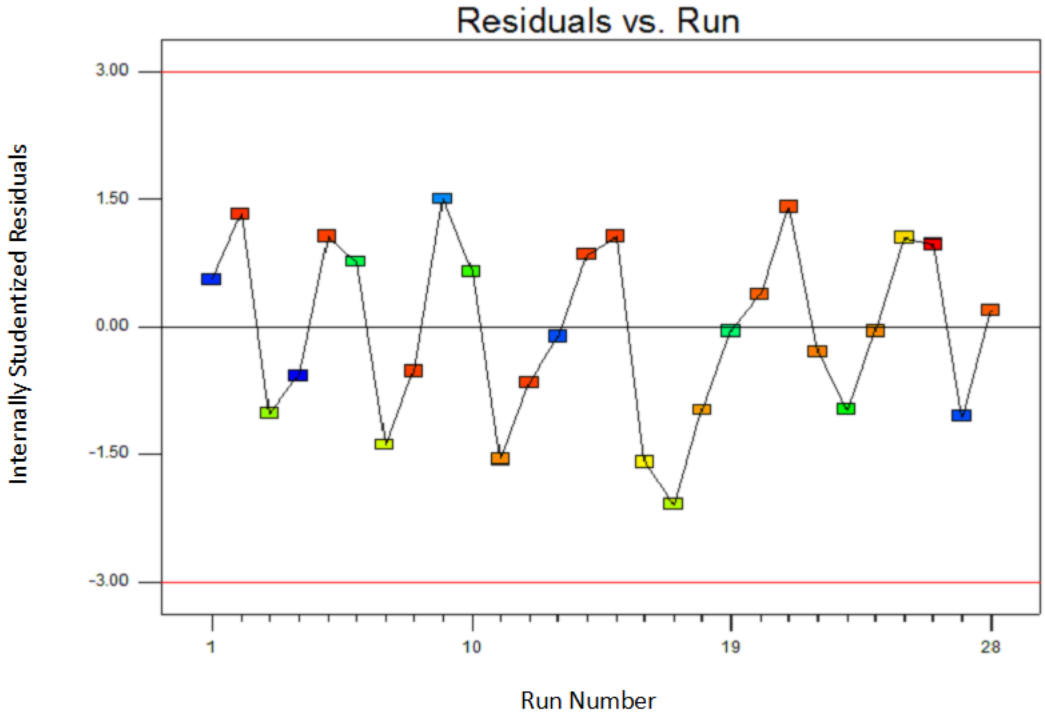

Figure S5. Residual plot of runs from central composite design for yield of *A. malaccensis* wood essential oil
